# Engineering the tumor microenvironment: oncolytic NDV to facilitate CAR-T cell therapy

**DOI:** 10.1186/s12967-025-07342-0

**Published:** 2025-11-19

**Authors:** Mei Wang, Ke Jiang, Alexandra Aicher, Christopher Heeschen

**Affiliations:** 1 Center for Single-Cell Omics, School of Public Health, Shanghai Jiao Tong University School of Medicine, Shanghai, China; 2 State Key Laboratory of Systems Medicine for Cancer, Shanghai Jiao Tong University School of Medicine, Shanghai, China; 3 Precision Immunotherapy, Graduate Institute of Biomedical Sciences, China Medical University, Taichung, Taiwan; 4 Pancreatic Cancer Heterogeneity, Candiolo Cancer Institute FPO-IRCCS, Candiolo, Turin, Italy

**Keywords:** Newcastle disease virus, Tumor microenvironment, CAR-T cell therapy, Oncolytic virus, Virotherapy, Immunotherapy, Immunomodulation

## Abstract

Chimeric antigen receptor (CAR)-T cell therapy has achieved significant progress in the treatment of hematologic cancers but continues to face major obstacles in solid tumors, including antigen heterogeneity, limited infiltration, and an immunosuppressive tumor microenvironment (TME). Oncolytic viruses (OVs) have emerged as promising tools to reshape the TME and improve CAR-T cell activity, yet many OVs encounter translational hurdles due to human seroprevalence and safety concerns. Newcastle disease virus (NDV), a naturally tumor-selective avian paramyxovirus, offers unique advantages as a non-integrating, non-pathogenic platform with a longstanding veterinary safety record and minimal pre-existing immunity in humans. NDV mediates direct oncolysis and immunogenic cell death, while simultaneously activating dendritic cells, repolarizing macrophages, and enhancing immune cell recruitment, thereby creating a TME that is more permissive to CAR-T cell therapy. Recent advances have enabled NDV to deliver immunostimulatory payloads locally within tumors, offering synergistic combinations to address CAR-T cell exhaustion and persistence. Looking ahead, further engineering efforts may expand the potential of this combined approach. This review summarizes the biological rationale, preclinical evidence, and translational prospects for integrating NDV with CAR-T cell therapy to improve outcomes in solid tumors.

## Highlights

Newcastle disease virus (NDV) offers low human seroprevalence and strong safety as an oncolytic platform.

NDV kills tumor cells and remodels the microenvironment to improve CAR-T cell efficacy.

Engineered NDV can deliver local cytokines, chemokines, and checkpoint inhibitors to overcome CAR-T cell exhaustion.

Synthetic biology may further expand NDV–CAR-T cell combination strategies.

## Introduction

### CAR-T cell therapy

is a form of adoptive immunotherapy that involves the genetic modification of T cells to recognize and attack cancer cells. CARs are engineered synthetic receptors that redirect lymphocytes, most commonly T cells, to recognize and eliminate cells expressing a specific target antigen. CAR-T cell therapy was developed to leverage the expansion, cytotoxicity and persistence of natural T cells while overcoming the major histocompatibility complex (MHC) restriction of T cell receptors (TCRs), thereby achieving broader therapeutic applicability [1]. Currently, CAR-T cell therapy has shown efficacy in patients with CD19-positive acute lymphoblastic leukemia and lymphoma. However, its application in solid tumors has been disappointing, mainly due to on-target off-tumor toxicity, physical barriers, and the highly immunosuppressive “cold” tumor microenvironment [2]. This persistent gap underscores the unmet need for approaches that can both remodel the tumor site and enhance CAR-T cell infiltration and persistence without adding systemic toxicity.

### The tumor microenvironment

(TME) in solid tumors plays a pivotal role not only in promoting cancer progression and metastasis but also in hindering immunotherapy efficacy. The TME is characterized by a complex network of suppressive immune cells, such as tumor-associated macrophages (TAMs), regulatory T cells (Tregs), and myeloid-derived suppressor cells (MDSCs), which creates a hostile environment for CAR-T cells and secretes immunosuppressive factors such as transforming growth factor-beta (TGF-β) and interleukin-10 (IL-10) [3, 4]. Additionally, the compact stromal cells and extracellular matrix (ECM) impair the trafficking and infiltration of CAR-T cells into the tumor core [5]. Furthermore, the loss or heterogeneity of tumor antigens and the upregulation of immune checkpoint molecules, such as PD-1/PD-L1, contribute to the suboptimal efficacy of CAR-T cells in solid tumors.

### To overcome the immunosuppressive TME,

multiple strategies have been investigated in both preclinical studies and clinical trials, including novel CAR structure modifications, combination immunotherapy, synthetic immunology approaches, nanomaterials and genetic engineering techniques [1, 6–8]. While these interventions have demonstrated partial therapeutic efficacy, they still face two critical limitations: an inability to comprehensively reverse the immunosuppressive nature of the TME, and the potential to induce significant systemic toxicities.

### Oncolytic Newcastle disease virus (NDV)

replicates and lyses in tumor cells while sparing normal cells, leading to direct oncolysis and induction of anti-cancer immune effects [9]. With the development of genetic engineering technology, NDV can also be genetically modified to deliver therapeutic transgenes locally in the TME, making it an appealing candidate for combination with CAR-T cell therapy [10]. Although NDV’s oncolytic effects and CAR-T cell engineering have been well-studied independently, research on their combined use is still scarce. Preliminary evidence suggests that NDV-mediated TME modulation could in theory enhance CAR-T cell function but mechanistic insights into this synergy are sparse. Specifically, how NDV could spatially and temporally augment CAR-T cell infiltration and persistence has yet to be systematically explored.

### This review

focuses on the concept that NDV, by reprogramming the TME, may transform the local immune landscape to enable effective CAR-T cell therapy in solid tumors. We further discuss engineering strategies to optimize NDV as a TME-modifying agent and outline future directions for integrating NDV and CAR-T cells into combined immunotherapy. Compared to other oncolytic viruses, NDV offers several unique advantages, including the absence of preexisting human immunity, broad tumor tropism through sialic acid binding, selective replication in apoptosis-resistant cancer cells, and excellent safety profiles from past clinical trials. These features make NDV a particularly promising platform to reshape the solid tumor microenvironment and enable CAR-T cell efficacy where other approaches have failed.

This review is based on publications retrieved from PubMed, Google Scholar, and ClinicalTrials.gov using combinations of the keywords “Newcastle disease virus,” “oncolytic virus,” “CAR-T cells,” “tumor microenvironment,” and “clinical trials.” Both classical NDV studies and recent advances in oncolytic virotherapy and CAR-T cell therapy were included, with preference given to original research articles and high-impact reviews published between 1957 and 2025.

## NDV basics

Oncolytic Newcastle Disease Virus (NDV) has been studied since the 1950s for its ability to selectively kill tumor cells [11]. Compared to other oncolytic viruses such as vaccinia virus, herpes simplex virus type 1 (HSV-1), adenovirus, measles virus, and reovirus, NDV offers several distinct advantages as an oncolytic agent.

**Table:** 

**Info box: why NDV (vs. HSV-1**, **vaccinia**, **reovirus**, **adenovirus) for CAR-T cell support?**
**Seroprevalence & redosing**. NDV has minimal pre-existing human immunity, enabling repeat dosing; adenovirus and vaccinia often face neutralization after the first dose.
**Tropism**. NDV engages sialic acids broadly expressed on tumors; reovirus relies on JAM-A, HSV-1 on HVEM/nectin-1, and adenovirus on the coxsackievirus and adenovirus receptor (encoded by *CXADR*) and integrins, narrowing eligible subpopulations.
**On-target TME effects**. NDV reliably triggers type-I IFN, immunogenic cell death, NK/DC/M1 polarization, and can be armored to deliver chemokines (e.g., CCL21; CCL19) or checkpoint scFvs intratumorally – ideal to address trafficking and exhaustion.
**Manufacturing & safety**. NDV is non-integrating, with decades of veterinary use and acceptable human safety across trials; established egg and Vero-cell platforms support scaling.
**Bottom line**. For CAR-T cell enablement (infiltration, persistence, exhaustion), NDV’s immuno-stimulatory profile and low seroprevalence make it a particularly suited TME-modifying partner.

### Seronegative profile

Unlike human-serotyped viruses (HSV-1, vaccinia) that face pre-existing immunity barriers [12, 13], NDV is an avian pathogen and does not naturally infect humans. In both preclinical studies (cynomolgus monkeys) and a Phase I clinical trial, baseline NDV neutralizing antibodies were undetectable, with only minimal increases after therapy, remaining within the normal control range [14]. NDV has been administered intravenously in most clinical studies, while subcutaneous and intradermal routes were used in earlier vaccine-based protocols [15, 16]. Intratumoral delivery is common in preclinical models, and recent interventional trials have also applied combined intravenous and intraperitoneal administration [14]. This stands in stark contrast to adenovirus vectors, which are hampered by high human seroprevalence [17]. The absence of pre-existing antibodies supports long-term and repeated NDV administration without rapid immune clearance [14]. Epidemiological studies confirm that approximately 96% of the human population is seronegative for NDV [10]. Interestingly, limited pre-existing immunity to NDV may even enhance systemic anti-tumor immune responses [18]. These findings emphasize NDV’s distinctive immunological profile and explain why it is considered a particularly promising partner for CAR-T cell therapy.

### Scalability

Most clinical trials to date have relied on egg-grown NDV, produced in chicken embryos [19]. A recent transition to mammalian cell culture systems such as Vero cells has improved therapeutic potency by reducing complement sensitivity. Viral particles grown in these systems can incorporate host proteins such as CD46, which shield them from complement attack and prolong their survival in human serum [20]. Beyond this, mammalian cell culture systems provide greater reproducibility, allow serum-free good manufacturing practices (GMP) production, and reduce batch-to-batch variability compared with egg-based methods. This shift in manufacturing technology is critical for large-scale clinical application, ensuring consistent viral quality, safety, and supply for future global use.

### Tumor targeting

#### Selective receptor interactions

**Fig. 1:**
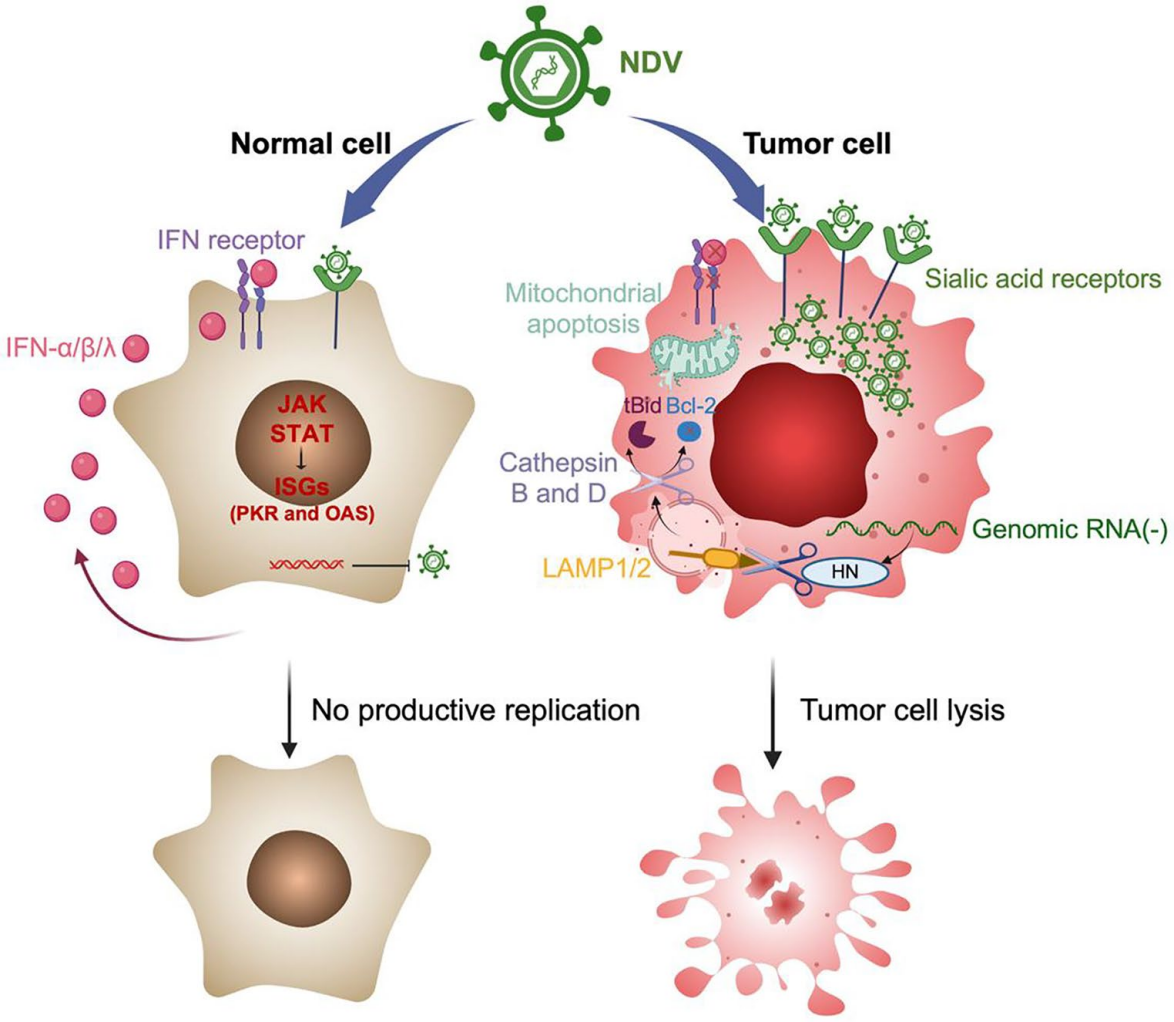
Newcastle Disease Virus preferentially replicates in tumor cells by exploiting defects in interferon signaling and apoptosis resistance. *NDV attaches to sialic acid residues that are present on both normal and tumor cells*,* with often vastly higher density seen on tumors (hypersialylation). In normal cells*,* infection induces intracellular production and secretion of type I (IFN-α/β) and type III (IFN-λ) that act in an autocrine/paracrine fashion to activate JAK–STAT and drive interferon-stimulated genes (ISGs) such as PKR and OAS*,* which block productive replication and limit cytotoxicity. Many tumor cells have weakened interferon signaling and resist classical apoptosis*,* allowing productive NDV replication. To complete its life cycle*,* NDV hemagglutinin-neuraminidase (HN) sialidase deglycosylates lysosomal membrane proteins LAMP1/2*,* destabilizing lysosomes and releasing cathepsins B and D. These enzymes convert pro-apoptotic Bid to its active form (tBid)*,* lower anti-apoptotic Bcl-2 levels*,* and trigger mitochondrial apoptosis*,* ensuring viral release and tumor cell destruction. Abbreviations: JAK–STAT*,* Janus kinase–signal transducer and activator of transcription; HN*,* hemagglutinin–neuraminidase; ISGs*,* interferon-stimulated genes; LAMP1/2*,* lysosomal-associated membrane protein 1/2; PKR*,* protein kinase R; OAS*,* 2’-5’-oligoadenylate synthetase; tBid*,* truncated BH3-interacting domain death agonist; Bcl-2*,* B-cell lymphoma 2. Created with* BioRender.com

NDV displays broad-spectrum oncolytic activity due to its preferential binding to sialic acid receptors, which are ubiquitously overexpressed across diverse human malignancies [21–23]. In contrast, many other oncolytic viruses require specific entry receptors that restrict their applicability. For instance, adenovirus relies on coxsackievirus and adenovirus receptors (encoded by *CXADR*) and integrins such as αvβ3 and αvβ5 for entry, whereas herpes simplex virus (HSV) requires herpesvirus entry mediator (HVEM) and nectin-1 [24, 25]. This biological constraint significantly limits their clinical applicability to defined patient subpopulations. Tumor cells frequently exhibit hypersialylation, characterized by increased expression of α2,3- and α2,6-linked sialic acid residues, which can enhance NDV binding (Fig. 1) [26]. Functional selectivity, however, arises from downstream effects and will be discussed in later sections. This broad tumor targeting is particularly advantageous when combining NDV with CAR-T cell therapy, as it allows for simultaneous viral debulking and microenvironment remodeling across a wide range of tumor types, regardless of CAR target heterogeneity.

#### Exploiting deficient antiviral defenses in tumors

NDV replication is further favored by defects in type I (IFN-α/β) and type III (IFN-λ) interferon signaling commonly found in tumor cells. The IFN-α/β and IFN-λ pathways normally activate JAK-STAT-mediated transcription of antiviral interferon-stimulated genes (*ISGs*), but tumors often disrupt these pathways through IFNAR1/2 downregulation, STAT1/2 mutations, or epigenetic silencing. This creates a permissive environment for NDV replication specifically in tumor cells, while sparing normal tissues. Even tumor cells with intact interferon signaling can sometimes be eliminated by NDV through alternative mechanisms such as direct oncolysis or immunogenic cell death (ICD) [27]. For CAR-T cell therapy, this tumor-selective NDV replication ensures that viral-mediated microenvironment modulation occurs preferentially at the tumor site, reducing off-target effects and making combined treatments safer and more focused.

#### Leveraging tumor-specific apoptosis resistance

Tumor cells frequently escape normal apoptosis through mutations in TP53, overexpression of anti-apoptotic proteins such as Bcl-2, Bcl-xL, or survivin, or loss of death receptor signaling via TRAIL-R or Fas [28]. While normal cells typically undergo rapid apoptosis upon viral infection, limiting viral spread, tumor cells’ resistance allows NDV to replicate and propagate effectively [29].

When replication is complete, NDV can actively force tumor cell death. One important mechanism involves lysosomal membrane permeabilization and cathepsin-mediated apoptosis, bypassing classical apoptotic resistance pathways [30]. Specifically, NDV’s HN protein deglycosylates the lysosomal membrane proteins LAMP1/2 through its sialidase activity, inducing lysosomal damage and the release of cathepsins B and D, which in turn cleave Bid, degrade Bcl-2, and activate mitochondrial apoptosis — effects shown to be suppressible with cathepsin inhibitors. Importantly, NDV can eliminate cancer cells when interferon responses are partially intact, highlighting its dual ability to exploit antiviral and apoptotic vulnerabilities. This ability to selectively kill apoptosis-resistant tumor cells is critical in CAR-T cell combinations, where NDV can debulk resistant tumor areas and break down physical and cellular barriers that otherwise limit CAR-T cell penetration and activity. Since apoptosis resistance is a hallmark of many cancers, including carcinomas, sarcomas, and hematologic malignancies [31, 32], this lysosomal targeting mechanism may be particularly valuable in therapy-resistant settings.

### Genetic engineering potential

NDV has a non-segmented, negative-sense single-stranded RNA genome with six essential genes: NP (nucleocapsid protein), P (phosphoprotein), M (matrix), F (fusion), HN (hemagglutinin-neuraminidase), and L (large polymerase), flanked by a 3’ leader and 5’ trailer, arranged 3’-NP-P-M-F-HN-L-5’, and separated by non-coding intergenic regions [33, 34]. This genome architecture, together with the paramyxovirus “rule-of-six”, supports insertion of therapeutic transgenes while maintaining proper transcription and replication. This modular organization allows insertion of therapeutic transgenes without disrupting native gene function.

Unlike DNA-based oncolytic vectors, NDV replicates entirely in the cytoplasm, thereby avoiding insertional mutagenesis [35]. It can stably accommodate foreign inserts up to ~ 3 kb, with studies showing robust expression of two transgenes without loss of viral fitness [36, 37]. Additionally, NDV exhibits exceptionally low homologous recombination rates, ensuring transgene stability even after repeated passaging [38]. Engineered NDV strains have delivered a wide range of immunomodulatory payloads demonstrating enhanced immune activation and improved anti-tumor efficacy across multiple preclinical models (summarized in Table 1). Anti-angiogenic payloads (e.g., anti-VEGFR2 scFv) have also been used to normalize tumor vasculature and reduce hypoxia, thereby enhancing radiosensitivity in NSCLC models [39].

For CAR-T cell combinations, NDV’s engineering potential enables local delivery of cytokines (e.g., IL-7/IL-15) to support persistence, chemokines (e.g., CCL19) to enhance trafficking, and intratumoral checkpoint blockade (e.g., PD-L1 scFv) to limit exhaustion – without systemic exposure, reducing toxicity and keeping the focus on CAR-T cell function [37, 39, 40]. This could render NDV not just a sole tumor debulking agent, but a programmable, locally acting immunomodulatory platform that can be tailored to address the specific bottlenecks limiting CAR-T cell efficacy in solid tumors.

**Table 1:** Engineered NDV and Immunomodulatory Effects on the TME

Transgene	Name	Impact on TME	Cancer Type	References
IL-2 + TRAIL	rNDV-IL-2-TRAIL	↑ CD8^+^ and CD4^+^ T cells	Hepatocellular carcinoma and melanoma	[41]
IL-2	NDV/Anh-IL-2	↑ CD8^+^ and CD4^+^ T cells	Hepatocellular carcinoma	[42]
P53	rNDV-P53	Regulates apoptotic signaling pathways	Glioblastoma, Hepatoma	[43]
IL-15 + IL-7	LX/IL-(15 + 7)	↑ CD8^+^ T; ↑IL-15, IL-7	Melanoma	[44]
IL-24	LX/IL-24	↑ CD8^+^ T	Melanoma	[45]
IL-12	rNDV-IL-12	↑ CD8^+^ T/NK cell infiltration	Breast cancer	[46]
anti-PD-1 scFv; anti-PD-L1 cFv; anti-CD28 super-agonist scFv; murine IL-12 fusions	rNDV-anti-PD-1; rNDV-anti-PD-L1; rNDV-anti-CD28; plus murine IL-12 fusion variants	↑IFN-α, granzyme B; ↑TILs	Melanoma	[37]
MIP-3α	NDV-MIP-3α	↑INF-γ-secreted CD8^+^ and CD4^+^ T cells;↓ CD25^+^ and FOXP3^+^; Attracts DCs	Melanoma and breast cancer	[47]
3α-LP	iNDV3α-LP	↑DAMPs; CD45^+^	Melanoma and breast cancer	[48]
OX40L	rNDV-mOX40L	↑ INF-γ; ↑CD4^+^ and CD8^+^ T cells	Colorectal cancer	[49]
GM-CSF	rNDV-GM-CSF (MEDI5395)	Upregulates CD80, PD-L1, HLA-DR and secretes IFN-α2a, IL-6, IL-8, TNF-α	Advanced solid tumors	[50]
αCTLA-4 + sPD-1	NDV-αCTLA-4 or NDV-sPD-1	Promotes stronger DC maturation and activation	Melanomas and colon carcinomas	[51]
IL-24 + GM-CSF	rClone30-IL-24-IRES-GM-CSF(P/M)	Reduces microangiogenesis; Recruits DCs and promotes their maturation;	Hepatocellular carcinoma	[52]
anti-VEGFR2 scFv	NDV-anti-VEGFR2	Normalizes tumor vasculature	Non-small cell lung cancer	[39]
GT	NDV-GT	↑CD4^+^ and CD8^+^ T cells; ↑Granzyme B and perforin release; ↑IL-2, TNF-α, IFN-γ and GM-CSF expression;	Multiple advanced cancers	[14]
human CCL19	rNDV19	↑IL-2, TNF-α and IFN-γ expression; ↑Granzyme A and perforin release; Recruit CAR-T cells into tumors;	Lung cancer	[40]

recombinant NDV, rNDV; IL-2, interleukin-2; IL-12, interleukin-12; IL-7, interleukin-7; IL-15, interleukin-15; IL-24, interleukin-24; PD-1, programmed cell death protein 1; PD-L1, programmed death-ligand 1; scFvs, single-chain variable fragments. NDV/Anh-IL-2, NDV Anhinga strain engineered to express human IL-2; iNDV3α-LP, iRGD-coated liposome encapsulating NDV expressing DC chemokine MIP-3α; LX/IL-(15 + 7), recombinant NDV LX strain engineered to express IL-15 and IL-7; GM-CSF, granulocyte-macrophage colony stimulating factor; GT, a1,3GT gene

## Mechanism of NDV in modulating the TME

Oncolytic NDV exerts its anti-tumor effect through two main mechanisms: direct oncolysis of cancer cells and immune-mediated tumor suppression.

### NDV’s direct effects on the TME

#### NDV-induced cell death

**Fig. 2:**
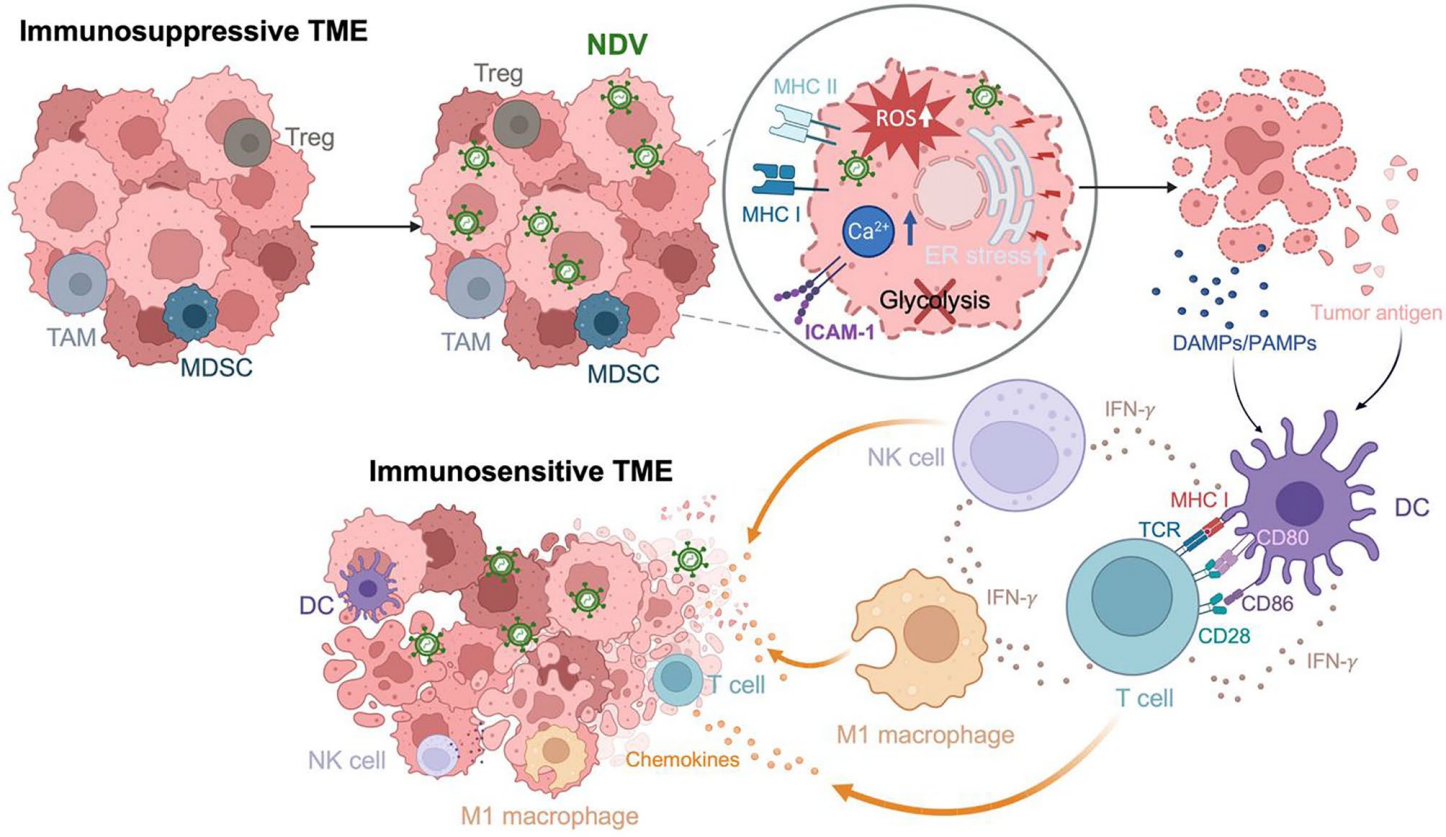
Mechanisms by which NDV remodels the TME. Oncolytic NDV exerts its anti-tumor effect through direct oncolysis of cancer cells and immune-mediated tumor suppression. NDV preferentially infects and replicates in tumor cells, producing progeny virions that disrupt cellular integrity. NDV induces mitochondrial damage and ER stress, perturbs glycolysis, and increases intracellular Ca^2+^ and reactive oxygen species (ROS), ultimately triggering tumor cell lysis. Oncolysis releases tumor antigen together with damage-associated molecular patterns (DAMPs) and pathogen-associated molecular patterns (PAMPs) that activate and mature DCs, increase antigen presentation, and upregulate co-stimulatory molecules such as CD80 and CD86. Type I interferons (IFN-α/β) and type III interferon (IFN-λ) produced by NDV-infected tumor cells increase MHC class I and adhesion molecules such as ICAM-1, enhancing immune recognition. Activated NK and T cells release IFN-γ, which augments antigen presentation, licenses DCs, and promotes macrophage polarization toward the M1 state. NDV-induced chemokines recruit NK cells, M1 macrophages, and T cells, collectively shifting the TME from immunosuppressive to immunosensitive. Created with BioRender.com

Following tumor-selective infection by NDV via binding to sialic-acid residues, attenuated interferon pathways, and apoptosis resistance, NDV replicates and produces progeny virions that disrupt cellular integrity, causing cytopathic lysis [53]. Additionally, the viral fusion (F) protein promotes membrane fusion and syncytia formation, further amplifying tumor cell destruction [54]. Despite the potent anti-apoptotic machinery of cancer cells, NDV induces mitochondrial damage and endoplasmic reticulum (ER) stress (Fig. 2) [55]. As such, unlike silent cell death, NDV triggers ICD, marked by calreticulin (CRT) exposure and DAMP release (e.g., HMGB1, ATP), effectively turning dying tumor cells into an “anticancer vaccine” and creating a bridge between oncolysis and immune activation [56, 57].

#### Metabolic TME remodeling

Cancer cells often upregulate glycolysis to meet their heightened energy demands and support rapid proliferation, a metabolic adaptation known as the Warburg effect [58]. Although glycolysis inhibition has shown therapeutic efficacy in many cancers, its benefits vary across tumor types due to metabolic heterogeneity [59]. NDV can induce multifaceted metabolic reprogramming within the TME. In breast cancer cells, NDV infection markedly suppressed hexokinase (HK) activity, decreases pyruvate and ATP levels, and reduces acidity, while sparing normal cells, indicating a substantial reduction in glycolytic activity in NDV-infected tumor cells [60]. Concurrently, NDV triggers Ca^2+^ accumulation and excessive ROS production, resulting in mitochondrial depolarization, loss of membrane potential, and the subsequent nuclear translocation of apoptosis-inducing factor (AIF), culminating in tumor cell death [61]. Importantly, some tumor subsets, such as pancreatic cancer stem cells, rely predominantly on oxidative phosphorylation (OXPHOS) [62, 63]. This underscores the need for context-specific evaluation of NDV-induced metabolic effects across cancer types.

**Table:** 

**Info box: NDV metabolism in PDAC**, **CSC caveats**, **and CAR-T cell timing**
**How other OVs use metabolism.** Among clinically used OVs, NDV is one of the few with primary evidence of directly dampening tumor glycolysis in vitro (reduced hexokinase activity, pyruvate, ATP, and acidity [60], whereas adenoviruses typically upshift glycolysis via E4ORF1–MYC [64], and vaccinia preferentially exploits non-glucose fuels such as glutamine anaplerosis and *de novo* lipogenesis [65].
**What NDV tends to do.** In some tumor models NDV reduces glycolytic readouts, remains active under hypoxia, and can lower HIF-1α. These shifts can lessen acidosis and support antitumor immunity.
**PDAC CSC caution.** Reliance on oxidative phosphorylation (OXPHOS) is enhanced in pancreatic cancer stem cells. If glycolysis is suppressed without limiting OXPHOS, these cells may persist. Consider context-specific combinations that include OXPHOS inhibition in selected settings.
**Implications for CAR-T cells.** Lower acidosis and hypoxia can improve T-cell trafficking, adhesion, and cytotoxicity. Sequence NDV before CAR-T cells, and avoid broad OXPHOS inhibition immediately after infusion when CAR-T cell expansion depends on OXPHOS-mediated mitochondrial fitness.
**Bottom line**. For CAR-T cell enablement (infiltration, persistence, exhaustion), NDV’s immuno-stimulatory profile and low seroprevalence make it a particularly suited TME-modifying partner.

Sustained tumor proliferation reprograms the TME, often creating hypoxia that can impair the replication of other oncolytic viruses such as adenovirus [66]. In contrast, NDV retains robust oncolytic activity under hypoxia and has been shown to lower hypoxia-induced HIF-1α accumulation, a pro-survival signal upregulated in many tumors [67].

Collectively, NDV-mediated metabolic reprogramming, which mitigates hypoxia and acidosis, together with ICD-mediated antigen release, may alleviate immunosuppressive barriers that limit CAR-T cell performance. By modulating the metabolic TME and enhancing antigen availability, NDV has the potential to improve CAR-T cell infiltration, persistence, and cytotoxicity, offering a promising strategy to overcome resistance in solid tumors.

### NDV-induced anti-tumor immune responses

While the oncolytic activity of NDV directly eliminates tumor cells and modulates the TME, NDV-induced cancer cell lysis further activates innate immune defenses, recruiting NK cells, dendritic cells, and macrophages. This not only enhances immediate immune clearance but also exposes neoantigens, which drives adaptive anti-tumor responses to overcome the immunosuppressive TME (Fig. 2).

#### NK cell activation

NDV effectively activates NK cells through multiple mechanisms. Its hemagglutinin-neuraminidase (HN) protein directly engages NK cell receptors such as NKp46, triggering cytotoxic degranulation, IFN-γ secretion, and upregulation of TRAIL via Syk/NF-κB signaling [68]. In parallel, NDV-induced type I interferons (IFN-α/β) further amplify NK cell activity by promoting TRAIL-mediated apoptosis of infected or malignant cells [69]. The contribution of NK cells to NDV’s anti-tumor effects has been demonstrated both in vitro and in vivo. For example, in pancreatic tumor models, NDV-driven NK cell activation was pivotal for initiating tumor rejection and establishing long-term anti-tumor immunity [70].

The NDV-mediated NK cell priming can create a “hot” TME primed for CAR-T cell therapy by reducing immunosuppressive barriers and increasing immune cell infiltration.

#### DC activation

In the immunosuppressive TME, DCs often become dysfunctional [71]. Restoring DC functionality is therefore a major goal in cancer immunotherapy. NDV-mediated tumor lysis releases viral RNA and previously hidden neoantigens which are taken up by local APCs, especially DCs, through surface and cytosolic pattern recognition receptors (PRRs) such as Toll-like receptors (TLR7/8) and RIG-I-like receptors (RIG-I, MDA5) [72, 73]. Engagement of these receptors triggers NF-κB and IRF3/IRF7 signaling, leading to the production of type I interferons and pro-inflammatory cytokines (e.g., TNF-α, IL-6, IL-8), ultimately enhancing DC cross-presentation [74].

Notably, even non-replicating NDV virus-like particles (VLPs) can activate DCs via the TLR4/NF-κB pathway, independent of viral replication [75]. This suggests that structural components of NDV are sufficient to initiate DC maturation. Furthermore, NDV-VLPs promote DC migration to lymph nodes, enhancing CD4^+^ T cell priming and boosting IFN-𝛾 and IL-4 production [75].

Together, these findings highlight NDV’s dual role in driving DC maturation and migration, effectively reprogramming the TME to support stronger adaptive immunity. In the context of CAR-T cell therapy, restoring and amplifying DC function may enhance endogenous immune responses, improve CAR-T cell recruitment, and increase overall anti-tumor efficacy.

#### Macrophage activation and polarization

Two independent laboratories, using different strains of NDV strains, reported that NDV strongly activates macrophages, endowing them with potent anti-tumor cytotoxic activity and mediating antimetastatic effects in vivo [76]. Within the TME, NDV infection promotes the production of pro-inflammatory cytokines such as IL-1α, TNF-α, IFN-γ, and IL-6 [77]. This inflammatory milieu drives macrophage polarization toward the M1 phenotype, which is associated with enhanced antigen presentation, tumor cell killing, and T cell recruitment. The M1 polarization is vital for reshaping the TME from a cold, immunosuppressive environment into one that supports robust anti-tumor immunity and improves the efficacy of immunotherapies, including CAR-T cells. By shifting the macrophage balance, NDV may help dismantle stromal barriers, enhance CAR-T cell infiltration, and provide local co-stimulatory signals that amplify CAR-T cell function.

### Potential roles of NDV-induced type I IFN and IFN-γ in CAR-T cell therapy

#### Type I interferons (IFN-β) and pro-inflammatory cytokines

NDV-induced IFN-I (notably IFN-β) upregulates HLA, HLA-DR, ICAM-1, and LFA-3, boosting immune recognition [78]. Since tumors often downregulate HLA and adhesion molecules to evade T cell attack [79, 80], NDV infection restores these markers, enhancing immune recognition and tumor-specific cytotoxicity [51, 81].

Beyond general immune effects, type I interferon signaling plays a complex, context-dependent role in CAR-T cell therapy [82, 83]. Prolonged or excessive type I IFN exposure upregulates PD-1 and TIM-3 on CAR-T cells, reducing their viability, and promoting resistance through PD-L1 upregulation in the TME [82, 84]. Importantly, NDV’s viral proteins, notably the V protein, can later suppress IFN-β production in a biphasic pattern [85, 86].This dynamic modulation of type I IFN defines a critical therapeutic window for combining NDV with CAR-T cell therapy: early IFN-β activation primes the TME and enhances immune recruitment, while later suppression or upregulation of immune checkpoints requires precise timing of CAR-T cell infusion to maximize anti-tumor effects and avoid immunosuppressive feedback [87].

Since type I IFN plays a dual role in both stimulating and suppressing CAR-T cell activity, titration strategies are crucial for balancing efficacy and exhaustion. Promoter engineering in NDV vectors can fine-tune type I IFN-related outputs by adjusting promoter strength or using inducible systems, which may sustain beneficial immune activation while limiting T cell exhaustion.

#### Interferon-γ

IFN-γ plays a key role in enhancing antigen presentation to activate endogenous T cells [81, 88]. While CAR-T cells do not require antigen presentation for tumor recognition, IFN-γ still indirectly supports CAR-T cell function by reshaping the TME, promoting M1 macrophage polarization, enhancing dendritic cell activation, and increasing local inflammatory cytokine production.

In solid tumor models, CAR-T cells themselves can further amplify IFN-γ signaling by activating host immune cells in the TME. However, IFN-γ’s role is highly context-dependent: In solid tumors, IFN-γ can upregulate ICAM-1 on target cells, strengthening the immunological synapse and enhancing CAR-T cell-mediated cytotoxicity [89, 90]. In some hematologic malignancies, however, IFN-γ blockade has been reported to improve CAR-T cell efficacy and reduce cytokine-related toxicities [91]. Furthermore, CAR-T cell responses to IFN-γ may vary by co-stimulatory domain; for example, CD28-based CAR-T cells have been reported as more susceptible to IFN-γ-induced apoptosis than 4-1BB-based CARs [92].

Although IFN-γ acts as a double-edged sword in CAR-T cell therapy, NDV’s capacity to boost local IFN-γ through innate and adaptive immune activation supports its use as an immunomodulatory partner in solid tumors, where pro-inflammatory conditioning of the TME may complement CAR-T cell strategies and help overcome the immunosuppressive barriers.

## Clinical & regulatory aspects

### Clinical development and regulatory divergence

In recent years, numerous clinical trials have explored the therapeutic potential of NDV (summarized in Table 2). Notably, NDV has shown activity across diverse tumor types and administration routes, with durable responses and minimal toxicity, reinforcing its safety profile and translational promise.

PV701, a naturally attenuated non-recombinant NDV strain, initially caused dose-limiting toxicities at doses exceeding 12 × 10⁹ PFU/m² due to rapid innate immune activation [93–95]. To mitigate this, a desensitization protocol consisting of a priming dose (1 × 10⁹ PFU/m²) followed 24 h later by a full dose (12 × 10⁹ PFU/m²), with extended infusion,reduced toxicity by more than 50% and allowed safe escalation to 120 × 10⁹ PFU/m² without accelerating neutralizingantibody formation [94, 95]. This underscores the importance of optimized dosing for safety and sustained efficacy.

**Table 2:** Selected clinical studies of oncolytic NDV in patients with cancer

NDV strain	Cancer type	Route	Outcome	Trial ID & Reference
73-T	Melanoma	Subcutaneous	55% 15-year overall survival; single-arm cohort	No trial ID [15]
ATV-NDV	Solid tumors	Intradermal	High disease control rates, with 2-year survival improvements of 20–36%	No trial ID [16]
MTH-68	High-grade glioblastomas	Intravenous	Long-term (> 5 years) survival and near-complete tumor regression in 4/4 terminal GBM patients, with no toxicity	No trial ID [96]
PV701	Advanced or recurrent tumors	Intravenous	20% ORR	NCT00081211 [93]
NDV-HUJ	Recurrent glioblastoma	Intravenous	One transient CR lasting > 3 months	NCT01174537 [97]
MEDI5395 (rNDV-GM-CSF)	Advanced solid tumors	Intravenous	10.3% ORR (PR) with manageable toxicity	NCT03889275 [50]
MEDI9253 (rNDV-IL 12)	Solid tumors	Intravenous	No efficacy data (ORR, PFS, OS) disclosed yet	NCT04613492 ^No reference yet^
NDV-GT	Multiple advanced cancers	Intravenous ± intraperitoneal	90% disease control (1 CR + 6 PR + 11 SD) with durable responses and no serious adverse events	ChiCTR2000031980 [14]

ATV-NDV, NDV-modified autologous tumor vaccine; ORR, objective response rate; PFS, progression-free survival; OS, overall survival; PR, partial response; CR, complete response; SD, stable disease

To address the limitations observed with early NDV programs such as PV701, contemporary efforts have shifted to genetically engineered NDV to enhance oncolytic potency and immunomodulation, thereby overcoming the constraints of natural NDV strains. Advances include MEDI5395 (rNDV-GM-CSF) and NDV-GT, which demonstrated immune activation with acceptable safety and tolerability. As noted earlier, NDV-GT exhibited robust anti-tumor immunity, achieving 90% disease control rate in refractory cancers and distant metastases, underscoring the potential of genetically modified oncolytic NDV in cancer immunotherapy [14].

Prospective patient selection may also enhance NDV outcomes: (i) Type-I IFN pathway attenuation (e.g., IFNAR/STAT alterations) predicts permissive replication [98]; (ii) tumor hypersialylation supports NDV binding [22]; (iii) TME state (baseline chemokines, M1/M2 ratio) and metabolic features (acidosis/hypoxia signatures) may indicate likelihood of NDV-mediated “hot-switching” [99, 100]; and (iv) CAR-T cell target stability plus PD-L1 dynamics inform the need for NDV-encoded checkpoint blockade [101]. Embedding these parameters in early trials using biopsies with single-cell and spatial profiling could accelerate rational NDV–CAR-T cell deployment.

Together, these developments indicate that progress depends not only on increasing potency but also on deliberately modulating host-virus interactions to convert immunological hurdles into therapeutic advantages, and selecting the most suitable patient cohort.

### Regulatory advantages of NDV over other oncolytic viruses

Beyond its biological advantages, NDV holds a distinct regulatory edge over many other oncolytic viruses. Notably, NDV has been safely used for decades as a live-attenuated vaccine in veterinary medicine to prevent Newcastle disease in poultry, with strains such as LaSota and B1 administered to millions of chickens worldwide. This long history has provided extensive data on NDV’s manufacturing, stability, environmental safety, and a favorable human safety profile, which lowers regulatory barriers when adapting it for human cancer therapy [102]. Moreover, NDV has been tested in multiple human cancer trials, consistently showing an exceptional safety profile with minimal toxicity (see Table 2). Importantly, NDV lacks detectable pre-existing immunity in humans, enabling repeated dosing without neutralization issues – unlike human-serotypic vectors such as adenovirus or vaccinia [10, 13] These combined features reduce regulatory hurdles for clinical translation, particularly when designing combinatorial approaches with CAR-T cell therapy, where safety concerns around viral delivery can otherwise slow development.

### Regulatory route

The transition of oncolytic virus therapies from bench to bedside involves navigating a well-defined regulatory pathway with specific ethical safeguards. In the U.S. and EU, talimogene laherparepvec (Imlygic) is the only approved oncolytic virus (FDA and EMA). Globally, additional OVs such as H101 in China [103] and teserpaturev G47Δ in Japan [104] have national approvals, which underscores that defined regulatory pathways for viral therapies exist [105, 106].

In the United States, NDV-based human therapies are regulated by the FDA’s Center for Biologics Evaluation and Research (CBER) as biological products, under the Public Health Service Act and Federal Food, Drug, and Cosmetic Act, with Investigational New Drug (IND) submissions providing Chemistry, Manufacturing, and Controls (CMC), nonclinical, and clinical information. Sponsors can rely on key product-class guidance documents, including: (i) CMC Information for Human Gene Therapy INDs; (ii) Long-Term Follow-Up After Administration of Human Gene Therapy Products; and (iii) Design and Analysis of Shedding Studies for Virus- or Bacteria-Based Gene Therapy and Oncolytic Products, along with FDA’s environmental assessment guidance for gene therapy and related recombinant viral/microbial products [107–109].

In the European Union, NDV products fall within the Advanced Therapy Medicinal Products (ATMP) framework, with gene-modified NDV classified as a Gene Therapy Medicinal Product (GTMP) under Regulation (EC) No 1394/2007 [110]. The EMA’s Committee for Advanced Therapies (CAT) provides ATMP classification advice and leads the scientific assessment within the centralized market authorization procedure [111]. For clinical trials involving genetically modified organisms (GMOs), EU Member States apply the EU GMO legislation, primarily Directive 2001/18/EC (deliberate release) and/or Directive 2009/41/EC (contained use), which run in parallel and provide a transparent biosafety evaluation alongside clinical trial authorization [112]. This GMO layer is separate from, and in addition to, the EMA/competent-authority medicinal product review.

Together, these U.S. and EU frameworks offer a clear and predictable route that covers CMC quality, viral shedding/transmission risk, environmental assessments, nonclinical/clinical study design, and long-term follow-up for gene-therapy/oncolytic products, positioning NDV—and future NDV–CAR-T cell combinations – for efficient, well-scaffolded development.

## Future perspective

### Engineering NDV for CAR-T cell support

**Fig. 3:**
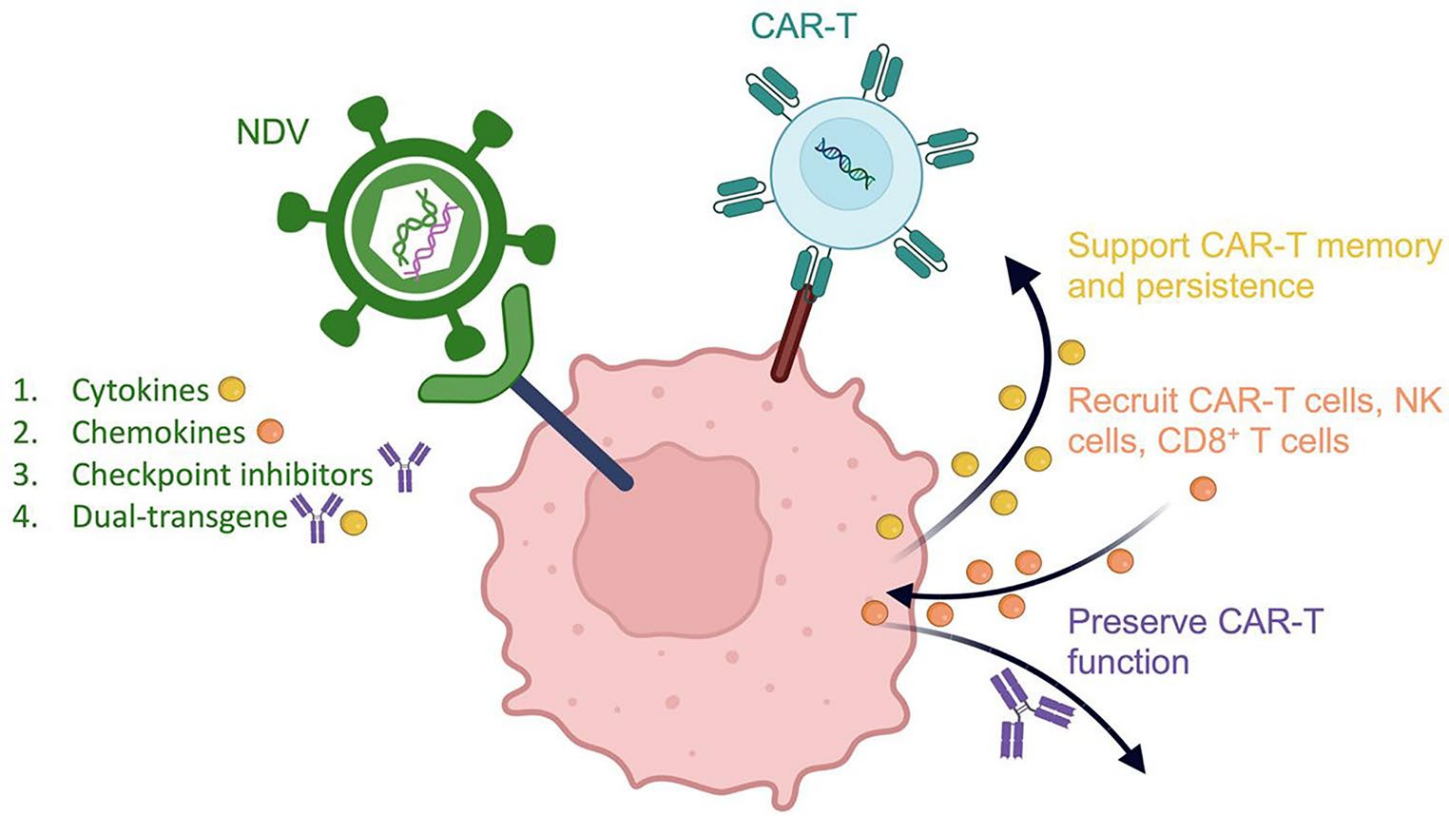
NDV–CAR-T cell combinations for solid tumors. Genetic engineering allows NDV to deliver immunomodulatory payloads within the TME: (1) cytokines (IL-2, IL-7, IL-12, IL-15) to support CAR-T cell memory and persistence; (2) chemokines (CCL21) to recruit CAR-T cells, NK cells, and CD8⁺ T cells, improving trafficking; (3) checkpoint inhibitors (anti-PD-1/anti-PD-L1 scFvs) to preserve CAR-T cell function and promote an inflamed TME; and (4) dual-transgene designs (e.g., IL-12 with anti-PD-1, or IL-2 with TRAIL) to activate and sustain anti-tumor immunity and remodel the immunosuppressive TME. Created with BioRender.com

In the evolving landscape of cancer immunotherapy, the combination of oncolytic viruses and CAR-T cell therapy has emerged as a promising strategy to enhance anti-tumor efficacy [113]. NDV holds particular potential as a carrier for delivering cytokines, chemokines, and checkpoint inhibitors to augment CAR-T cell function and persistence. This multifaceted approach leverages the unique properties of NDV to create a synergistic therapeutic environment within the immunosuppressive TME (Fig. 3).

#### Cytokines:

Cytokines such as IL-2, IL-12, IL-7, and IL-15 play pivotal roles in promoting CAR-T cell persistence, memory formation, and effector functions. IL-12, for instance, drives Th1 polarization, which is crucial for robust anti-tumor immunity, and has been shown to enhance CAR-T cell persistence when locally delivered. One study demonstrated that systemic administration of recombinant NDV expressing IL-12 (rNDV-IL-12) alone provided limited anti-tumor activity. However, when combined with HER2-targeted CAR-T cells, it significantly prolonged survival and reversed T cell exhaustion [114]. IL-7 and IL-15 support memory CD8⁺ T cells and the combined action of IL-7 and IL-15 is crucial for enhancing CAR-T cell efficacy [115]. The success of IL-7 and IL-15-armored CAR-T cells validates their critical roles in sustaining T cell memory and preventing T cell exhaustion in TME [116, 117]. The engineering of NDV to deliver IL-7 and IL-15 presents a clinically viable strategy to enhance CAR-T cell memory formation and persistence within solid tumors. Engineering NDV to express these cytokines allows sustained local release within the TME, which may maximize CAR-T cell benefits while minimizing systemic toxicity.

#### Chemokines:

Chemokines play a key role in enhancing immune cell recruitment to the tumor site [118]. By attracting effector cells, chemokines help convert an immunologically “cold” TME into a “hot” one, creating a supportive immune context that enhances CAR-T cell trafficking and complements endogenous anti-tumor immunity. A recent study demonstrated the potential of a CCL19-expressing recombinant NDV (rNDV19) to boost CAR-T cell infiltration and efficacy in solid tumors. This research highlights that rNDV19 not only increases the local concentration of CCL19 within the TME but also promotes the migration and activation of T cells, including CAR-T cells, increasing local CCL19 levels and promoting T cell migration and activation in preclinical models [40]. NDV, as a viral carrier, offers an ideal platform for local chemokine delivery to overcome immune exclusion in solid tumors. Engineering NDV to express T cell-recruiting chemokines (e.g., CCL21) could improve CAR-T trafficking, strengthen anti-tumor responses, and enhance CAR-T cell efficacy [119].

#### Checkpoint inhibitors:

The interaction between PD-1 on T cells and PD-L1 on tumor and stromal cells is a well-documented mechanism of immune evasion [120]. PD-1 and PD-L1 interactions drive T cell dysfunction and exhaustion, which significantly impair CAR-T cell function in the TME [121]. Checkpoint inhibitors, including anti-PD-1 or anti-PD-L1 antibodies and their scFv formats, can effectively block this interaction, preserving CAR-T cell function within the immunosuppressive TME [121, 122]. While CAR-T-secreted PD-1-TREM2 scFv demonstrates the promise of localized checkpoint blockade targeting both T cell and myeloid checkpoints, its efficacy remains intrinsically tied to CAR-T cell viability and infiltration [123]. Engineering NDV to express these inhibitors enables localized, sustained intratumoral blockade of PD-1/PD-L1, potentially reducing systemic toxicities while enhancing therapeutic efficacy [37, 51]. This strategy can both prolong CAR-T cell persistence and recondition the TME toward a more inflamed, treatment-permissive state, supporting broader and more durable anti-tumor responses.

#### Dual-transgene NDV vectors:

Dual-transgene strategies simultaneously activate and sustain anti-tumor immunity within the TME. Preclinical studies demonstrate that IL-12 primes a pro-inflammatory milieu by driving Th1 polarization and IFN-γ production, while PD-1/PD-L1 blockade counters T-cell exhaustion. In PD-L1-high models, recombinant NDV (rNDV) expressing IL-12 together with anti-PD-1/PD-L1 scFv produced stronger tumor regression than IL-12 alone, which was limited by adaptive PD-L1 upregulation. These vectors also reduced MDSCs/Tregs and increased functional CD8^+^ T cell infiltration, with activity further improved by systemic anti-CTLA-4 in bilateral tumors [37]. Separately, an rNDV co-expressing IL-2 and TRAIL (rNDV-IL-2-TRAIL) combined TRAIL-mediated apoptosis with IL-2-driven T-cell activation, significantly slowing tumor progression [41]. Together, these dual-modality designs address two recurring barriers in solid tumors – immunosuppression and poor T-cell persistence – and suggest a path to more permissive TMEs for future CAR-T cell combinations.

The use of NDV as a versatile delivery platform for cytokines, chemokines, and checkpoint inhibitors represents a clinically promising strategy to overcome the limitations of CAR-T cell therapy in solid tumors. With established biosafety profiles in human trials and scalable manufacturing processes already implemented in vaccine production, this integrated approach not only augments the direct cytotoxic effects of NDV but also enhances the functionality and persistence of CAR-T cells, offering a powerful new direction in the treatment of cancer.

### Next-generation NDV–CAR-T cell platforms

The next wave of cancer immunotherapy will move beyond static CAR-T cells and oncolytic viruses, leveraging synthetic biology to create adaptive, self-organizing systems in which NDV and CAR-T cells collaborate dynamically within the TME. Synthetic biology approaches could enable programmable NDV “immune circuits” that sense TME conditions in real time, releasing tailored payloads only when CAR-T cells are present or when specific resistance mechanisms are detected. Tumor cells often evade immune detection by downregulating antigen presentation [124]. It may even become feasible to engineer NDV to encode transient, tumor-restricted “decoy antigens,” allowing CAR-T cells to dynamically retarget themselves as tumors evolve without the need to redesign the CAR construct [125, 126].

In parallel, synthetic Notch (synNotch)-engineered CAR-T cells could be designed to recognize NDV-induced tumor antigens and, upon detection, trigger programmable downstream responses, such as enhanced cytotoxicity, localized cytokine release, or phenotypic adaptation [127–129]. These multilayered, conditional strategies enable unprecedented precision, allowing immune cells and oncolytic viruses to function as a coordinated, self-regulating therapeutic system. While these ideas remain speculative, such approaches demonstrate the transformative potential of combining viral oncolysis, gene delivery, and synthetic immunology for next-generation cancer immunotherapy.

### Conclusion

NDV offers several advantages over other oncolytic platforms for supporting CAR-T cell therapy. Its natural tumor selectivity ensures a high safety margin, while its seronegative profile in humans allows long-term, repeated systemic dosing without neutralization. NDV is a potent inducer of type I interferon and chemokine networks that counteract immune exclusion and establish favorable conditions for CAR-T cell trafficking and activation. NDV administration prior to CAR-T infusion reduces tumor burden and matrix density, promotes vascular remodeling, and induces an interferon-driven inflammatory milieu that facilitates effector cell entry. Engineered NDV variants can further strengthen this synergy by expressing cytokines or chemokines such as IL-15, CCL19, and CCL21, thereby sustaining CAR-T cell persistence and enhancing intratumoral recruitment. These combined features position NDV as a clinically compatible and versatile platform to enhance the infiltration, persistence, and overall efficacy of CAR-T cells in solid tumors.

## Data Availability

Not applicable. No new datasets were generated or analyzed. All data discussed are from previously published sources cited in the manuscript.

## Declarations

### Writing and review of the manuscript

M.W., K.J., A.A., C.H.

## References

[1] Du B, Qin J, Lin B, Zhang J, Li D, Liu M. CAR-T therapy in solid tumors. Cancer Cell. 2025;43(4):665–79. 40233718 10.1016/j.ccell.2025.03.019

[2] Albelda SM. CAR T cell therapy for patients with solid tumours: key lessons to learn and unlearn. Nat Reviews Clin Oncol. 2024;21(1):47–66. 10.1038/s41571-023-00832-4 37904019

[3] Peng JJ, Wang L, Li Z, Ku CL, Ho PC. Metabolic challenges and interventions in CAR T cell therapy. Sci Immunol. 2023;8(82):eabq3016. 37058548 10.1126/sciimmunol.abq3016

[4] Albelda SM. CAR T cell therapy for patients with solid tumours: key lessons to learn and unlearn. Nat Rev Clin Oncol. 2024;21(1):47–66. 37904019 10.1038/s41571-023-00832-4

[5] Mai Z, Lin Y, Lin P, Zhao X, Cui L. Modulating extracellular matrix stiffness: a strategic approach to boost cancer immunotherapy. Cell Death Dis. 2024;15(5):307. 38693104 10.1038/s41419-024-06697-4 PMC11063215

[6] Rafiq S, Hackett CS, Brentjens RJ. Engineering strategies to overcome the current roadblocks in CAR T cell therapy. Nat Reviews Clin Oncol. 2020;17(3):147–67. 10.1038/s41571-019-0297-y PMC7223338 31848460

[7] Hu B, Xiao X, Chen P, et al. Enhancing anti-tumor effect of ultrasensitive bimetallic RuCu nanoparticles as radiosensitizers with dual enzyme-like activities. Biomaterials. 2022;290:121811. 36201948 10.1016/j.biomaterials.2022.121811

[8] Xu B, Cui Y, Wang W, et al. Immunomodulation-Enhanced Nanozyme-Based tumor catalytic therapy. Adv Mater. 2020;32(33):e2003563. 32627937 10.1002/adma.202003563

[9] Schirrmacher V, van Gool S, Stuecker W. Breaking therapy resistance: an update on oncolytic newcastle disease virus for improvements of cancer therapy. Biomedicines. 2019;7(3). 10.3390/biomedicines7030066 PMC6783952 31480379

[10] Zamarin D, Palese P. Oncolytic Newcastle disease virus for cancer therapy: old challenges and new directions. Future Microbiol. 2012;7(3):347–67. 22393889 10.2217/fmb.12.4 PMC4241685

[11] Prince AM, Ginsberg HS. Studies on the cytotoxic effect of Newcastle disease virus (NDV) on Ehrlich Ascites tumor cells. I. Characteristics of the virus-cell interaction. J Immunol. 1957;79(2):94–106. 13475814

[12] Harfouche M, AlMukdad S, Alareeki A, et al. Estimated global and regional incidence and prevalence of herpes simplex virus infections and genital ulcer disease in 2020: mathematical modelling analyses. Sex Transm Infect. 2025;101(4):214–23. 39658199 10.1136/sextrans-2024-056307 PMC12128767

[13] Hsu J, Kim S, Anandasabapathy N. Vaccinia virus: mechanisms supporting immune evasion and successful long-term protective immunity. Viruses. 2024;16(6). 10.3390/v16060870 PMC11209207 38932162

[14] Zhong L, Gan L, Wang B, et al. Hyperacute rejection-engineered oncolytic virus for interventional clinical trial in refractory cancer patients. Cell. 2025;188(4):1119–e3623. 39826543 10.1016/j.cell.2024.12.010

[15] Batliwalla FM, Bateman BA, Serrano D, et al. A 15-year follow-up of AJCC stage III malignant melanoma patients treated postsurgically with Newcastle disease virus (NDV) oncolysate and determination of alterations in the CD8 T cell repertoire. Mol Med. 1998;4(12):783–94. 9990864 PMC2230393

[16] Schirrmacher V. Clinical trials of antitumor vaccination with an autologous tumor cell vaccine modified by virus infection: improvement of patient survival based on improved antitumor immune memory. Cancer Immunol Immunother. 2005;54(6):587–98. 15838708 10.1007/s00262-004-0602-0 PMC11042470

[17] Hong L, Li J, Zeng W, et al. The Seroprevalence of adenoviruses since 2000(1). Emerg Microbes Infect. 2025;14(1):2475831. 40035700 10.1080/22221751.2025.2475831 PMC11915735

[18] Ricca JM, Oseledchyk A, Walther T, et al. Pre-existing immunity to oncolytic virus potentiates its immunotherapeutic efficacy. Mol Therapy: J Am Soc Gene Therapy. 2018;26(4):1008–19. 10.1016/j.ymthe.2018.01.019 PMC6079372 29478729

[19] Biswas M, Johnson JB, Kumar SR, Parks GD, Elankumarana S. Incorporation of host complement regulatory proteins into Newcastle disease virus enhances complement evasion. J Virol. 2012;86(23):12708–16. 22973037 10.1128/JVI.00886-12 PMC3497656

[20] Huberts M, de Graaf JF, Groeneveld D, van Nieuwkoop S, Fouchier RAM, van den Hoogen BG. Cell-derived Newcastle disease virus variant with two amino acid substitutions near cleavage site of F shows favorable traits as oncolytic virus. Mol Ther Oncol. 2025;33(1):200915. 39802675 10.1016/j.omton.2024.200915 PMC11719830

[21] Rodriguez E, Boelaars K, Brown K, et al. Sialic acids in pancreatic cancer cells drive tumour-associated macrophage differentiation via the Siglec receptors Siglec-7 and Siglec-9. Nat Commun. 2021;12(1):1270. 33627655 10.1038/s41467-021-21550-4 PMC7904912

[22] Li Q, Wei D, Feng F, et al. alpha2,6-linked Sialic acid serves as a high-affinity receptor for cancer oncolytic virotherapy with Newcastle disease virus. J Cancer Res Clin Oncol. 2017;143(11):2171–81. 28687873 10.1007/s00432-017-2470-y PMC11819098

[23] Ghosh M, Hazarika P, Dhanya SJ, Pooja D, Kulhari H. Exploration of Sialic acid receptors as a potential target for cancer treatment: A comprehensive review. Int J Biol Macromol. 2024;257(Pt 1):128415. 38029891 10.1016/j.ijbiomac.2023.128415

[24] Agarwal P, Gammon EA, Sajib AM, Sandey M, Smith BF. Cell-Surface integrins and CAR are both essential for adenovirus type 5 transduction of canine cells of lymphocytic origin. PLoS ONE. 2017;12(1):e0169532. 28068367 10.1371/journal.pone.0169532 PMC5222425

[25] Madavaraju K, Koganti R, Volety I, Yadavalli T, Shukla D. Herpes simplex virus cell entry mechanisms: an update. Front Cell Infect Microbiol. 2020;10:617578. 33537244 10.3389/fcimb.2020.617578 PMC7848091

[26] Dong B, Tang N, Guan Y, et al. Type and abundance of Sialic acid receptors on host cell membrane affect infectivity and viral titer of different strains of Newcastle disease virus. J Virol Methods. 2022;302:114488. 35108596 10.1016/j.jviromet.2022.114488

[27] Ginting TE, Christian S, Larasati YO, Suryatenggara J, Suriapranata IM, Mathew G. Antiviral interferons induced by Newcastle disease virus (NDV) drive a tumor-selective apoptosis. Sci Rep. 2019;9(1):15160. 31641164 10.1038/s41598-019-51465-6 PMC6806003

[28] Hanahan D. Hallmarks of cancer: new dimensions. Cancer Discov. 2022;12(1):31–46. 35022204 10.1158/2159-8290.CD-21-1059

[29] Mansour M, Palese P, Zamarin D. Oncolytic specificity of Newcastle disease virus is mediated by selectivity for apoptosis-resistant cells. J Virol. 2011;85(12):6015–23. 21471241 10.1128/JVI.01537-10 PMC3126310

[30] Chen Y, Zhu S, Liao T, et al. The HN protein of Newcastle disease virus induces cell apoptosis through the induction of lysosomal membrane permeabilization. PLoS Pathog. 2024;20(2):e1011981. 38354122 10.1371/journal.ppat.1011981 PMC10866534

[31] Carneiro BA, El-Deiry WS. Targeting apoptosis in cancer therapy. Nat Rev Clin Oncol. 2020;17(7):395–417. 32203277 10.1038/s41571-020-0341-y PMC8211386

[32] Shahar N, Larisch S. Inhibiting the inhibitors: targeting anti-apoptotic proteins in cancer and therapy resistance. Drug Resist Updat. 2020;52:100712. 32599435 10.1016/j.drup.2020.100712

[33] Yusoff K, Tan WS. Newcastle disease virus: macromolecules and opportunities. Avian Pathol. 2001;30(5):439–55. 19184932 10.1080/03079450120078626

[34] Elbehairy MA, Samal SK, Belov GA. Encoding of a transgene in-frame with a newcastle disease virus protein increases transgene expression and stability. J Gen Virol. 2022;103(6). 10.1099/jgv.0.001761 PMC10027024 35758932

[35] Bukreyev A, Skiadopoulos MH, Murphy BR, Collins PL. Nonsegmented negative-strand viruses as vaccine vectors. J Virol. 2006;80(21):10293–306. 17041210 10.1128/JVI.00919-06 PMC1641758

[36] He L, Zhang Z, Yu Q. Expression of two foreign genes by a Newcastle disease virus vector from the optimal insertion sites through a combination of the ITU and IRES-Dependent expression approaches. Front Microbiol. 2020;11:769. 32411112 10.3389/fmicb.2020.00769 PMC7198723

[37] Vijayakumar G, McCroskery S, Palese P. Engineering Newcastle disease virus as an oncolytic vector for intratumoral delivery of immune checkpoint inhibitors and immunocytokines. J Virol. 2020;94(3). 10.1128/JVI.01677-19 PMC7000961 31694938

[38] Engel-Herbert I, Werner O, Teifke JP, Mebatsion T, Mettenleiter TC, Römer-Oberdörfer A. Characterization of a Recombinant Newcastle disease virus expressing the green fluorescent protein. J Virol Methods. 2003;108(1):19–28. 12565150 10.1016/s0166-0934(02)00247-1

[39] Liu L, Song L, Liu T et al. Recombinant oncolytic virus NDV-anti-VEGFR2 enhances radiotherapy sensitivity in NSCLC by targeting VEGF signaling and impairing DNA repair. Gene therapy. 2025. 10.1038/s41434-025-00540-x 40382521

[40] Liu M, Li C, Feng LT et al. hCCL19-expressing recombinant newcastle disease virus boosts CAR T cell infiltration and efficacy in solid tumor. J Immunother Cancer. 2025;13(7). 10.1136/jitc-2025-011783 PMC12306363 40713180

[41] Bai FL, Yu YH, Tian H, et al. Genetically engineered Newcastle disease virus expressing interleukin-2 and TNF-related apoptosis-inducing ligand for cancer therapy. Cancer Biol Ther. 2014;15(9):1226–38. 24971746 10.4161/cbt.29686 PMC4128865

[42] Wu Y, He J, An Y, et al. Recombinant Newcastle disease virus (NDV/Anh-IL-2) expressing human IL-2 as a potential candidate for suppresses growth of hepatoma therapy. J Pharmacol Sci. 2016;132(1):24–30. 27174862 10.1016/j.jphs.2016.03.012

[43] An Y, Liu T, He J, et al. Recombinant Newcastle disease virus expressing P53 demonstrates promising antitumor efficiency in hepatoma model. J Biomed Sci. 2016;23(1):55. 27465066 10.1186/s12929-016-0273-0 PMC4964062

[44] Xu X, Sun Q, Mei Y, Liu Y, Zhao L. Newcastle disease virus co-expressing Interleukin 7 and Interleukin 15 modified tumor cells as a vaccine for cancer immunotherapy. Cancer Sci. 2018;109(2):279–88. 29224228 10.1111/cas.13468 PMC5797827

[45] Xu X, Yi C, Yang X, et al. Tumor cells modified with Newcastle disease virus expressing IL-24 as a cancer vaccine. Mol Ther Oncolytics. 2019;14:213–21. 31338417 10.1016/j.omto.2019.06.001 PMC6630061

[46] Mohamed Amin Z, Che Ani MA, Tan SW, et al. Evaluation of a Recombinant Newcastle disease virus expressing human IL12 against human breast cancer. Sci Rep. 2019;9(1):13999. 31570732 10.1038/s41598-019-50222-z PMC6768883

[47] Huang FY, Wang JY, Dai SZ et al. A recombinant oncolytic newcastle virus expressing MIP-3alpha promotes systemic antitumor immunity. J Immunother Cancer. 2020;8(2). 10.1136/jitc-2019-000330 PMC7410001 32759233

[48] Wang JY, Chen H, Dai SZ et al. Immunotherapy combining tumor and endothelium cell lysis with immune enforcement by recombinant MIP-3alpha newcastle disease virus in a vessel-targeting liposome enhances antitumor immunity. J Immunother Cancer. 2022;10(3). 10.1136/jitc-2021-003950 PMC8905871 35256516

[49] Tian L, Liu T, Jiang S, et al. Oncolytic Newcastle disease virus expressing the co-stimulator OX40L as immunopotentiator for colorectal cancer therapy. Gene Ther. 2023;30(1–2):64–74. 34602608 10.1038/s41434-021-00256-8

[50] Davar D, Carneiro BA, Dy GK et al. Phase I study of a recombinant attenuated oncolytic virus, MEDI5395 (NDV-GM-CSF), administered systemically in combination with durvalumab in patients with advanced solid tumors. J Immunother Cancer. 2024;12(11). 10.1136/jitc-2024-009336 PMC11574399 39551600

[51] Santry LA, van Vloten JP, AuYeung AWK, et al. Recombinant Newcastle disease viruses expressing immunological checkpoint inhibitors induce a pro-inflammatory state and enhance tumor-specific immune responses in two murine models of cancer. Front Microbiol. 2024;15:1325558. 38328418 10.3389/fmicb.2024.1325558 PMC10847535

[52] Wu Q, Jin Y, Li S, et al. Oncolytic Newcastle disease virus carrying the IL24 gene exerts antitumor effects by inhibiting tumor growth and vascular sprouting. Int Immunopharmacol. 2024;136:112305. 38823178 10.1016/j.intimp.2024.112305

[53] Ravindra PV, Tiwari AK, Ratta B, Chaturvedi U, Palia SK, Chauhan RS. Newcastle disease virus-induced cytopathic effect in infected cells is caused by apoptosis. Virus Res. 2009;141(1):13–20. 19152817 10.1016/j.virusres.2008.12.008

[54] Ren S, Rehman ZU, Shi M, et al. Syncytia generated by hemagglutinin-neuraminidase and fusion proteins of virulent Newcastle disease virus induce complete autophagy by activating AMPK-mTORC1-ULK1 signaling>. Vet Microbiol. 2019;230:283–90. 30658866 10.1016/j.vetmic.2019.01.002

[55] Elankumaran S, Rockemann D, Samal SK. Newcastle disease virus exerts Oncolysis by both intrinsic and extrinsic caspase-dependent pathways of cell death. J Virol. 2006;80(15):7522–34. 16840332 10.1128/JVI.00241-06 PMC1563725

[56] Koks CA, Garg AD, Ehrhardt M, et al. Newcastle disease virotherapy induces long-term survival and tumor-specific immune memory in orthotopic glioma through the induction of Immunogenic cell death. Int J Cancer. 2015;136(5):E313–25. 25208916 10.1002/ijc.29202

[57] Peng J, Li S, Ti H. Sensitize tumor immunotherapy: Immunogenic cell death inducing nanosystems. Int J Nanomed. 2024;19:5895–930. 10.2147/IJN.S457782 PMC11184231 38895146

[58] Liberti MV, Locasale JW. Correction to: ‘The Warburg effect: how does it benefit cancer Cells?‘: [Trends in biochemical Sciences, 41 (2016) 211]. Trends Biochem Sci. 2016;41(3):287. 29482833 10.1016/j.tibs.2016.01.004

[59] Yu TJ, Ma D, Liu YY, et al. Bulk and single-cell transcriptome profiling reveal the metabolic heterogeneity in human breast cancers. Mol Therapy: J Am Soc Gene Therapy. 2021;29(7):2350–65. 10.1016/j.ymthe.2021.03.003 PMC8261089 33677091

[60] Al-Ziaydi AG, Al-Shammari AM, Hamzah MI, Kadhim HS, Jabir MS. Newcastle disease virus suppress Glycolysis pathway and induce breast cancer cells death. Virusdisease. 2020;31(3):341–8. 32904847 10.1007/s13337-020-00612-z PMC7458979

[61] Qu Y, Wang S, Jiang H, et al. Newcastle disease virus infection induces parthanatos in tumor cells via calcium waves. PLoS Pathog. 2024;20(12):e1012737. 39621796 10.1371/journal.ppat.1012737 PMC11637436

[62] Keoh LQ, Chiu C-F, Ramasamy TS. Metabolic plasticity and cancer stem cell metabolism: exploring the glycolysis-OXPHOS switch as a mechanism for resistance and tumorigenesis. Stem Cell Reviews and Reports. 2025. 10.1007/s12015-025-10956-y PMC12504402 40880049

[63] Niu N, Shen X, Wang Z, et al. Tumor cell-intrinsic epigenetic dysregulation shapes cancer-associated fibroblasts heterogeneity to metabolically support pancreatic cancer. Cancer Cell. 2024;42(5):869–84. e9. 38579725 10.1016/j.ccell.2024.03.005

[64] Thai M, Graham NA, Braas D, et al. Adenovirus E4ORF1-induced MYC activation promotes host cell anabolic glucose metabolism and virus replication. Cell Metab. 2014;19(4):694–701. 24703700 10.1016/j.cmet.2014.03.009 PMC4294542

[65] Fontaine KA, Camarda R, Lagunoff M. Vaccinia virus requires glutamine but not glucose for efficient replication. J Virol. 2014;88(8):4366–74. 24501408 10.1128/JVI.03134-13 PMC3993723

[66] Huang R, Huestis M, Gan ES, Ooi EE, Ohh M. Hypoxia and viral infectious diseases. JCI Insight. 2021;6(7). 10.1172/jci.insight.147190 PMC8119216 33830079

[67] Ch’ng WC, Stanbridge EJ, Yusoff K, Shafee N. The oncolytic activity of Newcastle disease virus in clear cell renal carcinoma cells in normoxic and hypoxic conditions: the interplay between von Hippel-Lindau and interferon-beta signaling. J Interferon Cytokine Res. 2013;33(7):346–54. 23506478 10.1089/jir.2012.0095 PMC3708626

[68] Jarahian M, Watzl C, Fournier P, et al. Activation of natural killer cells by Newcastle disease virus hemagglutinin-neuraminidase. J Virol. 2009;83(16):8108–21. 19515783 10.1128/JVI.00211-09 PMC2715740

[69] Huang J, Zheng T, Liang Y, Qin Y, Wu X, Fan X. Transcriptome analysis of natural killer cells in response to Newcastle disease virus infected hepatocellular carcinoma cells. Genes (Basel). 2023;14(4). 10.3390/genes14040888 PMC10138298 37107646

[70] Schwaiger T, Knittler MR, Grund C, et al. Newcastle disease virus mediates pancreatic tumor rejection via NK cell activation and prevents cancer relapse by prompting adaptive immunity. Int J Cancer. 2017;141(12):2505–16. 28857157 10.1002/ijc.31026

[71] Zhu S, Yang N, Wu J, et al. Tumor microenvironment-related dendritic cell deficiency: a target to enhance tumor immunotherapy. Pharmacol Res. 2020;159:104980. 32504832 10.1016/j.phrs.2020.104980

[72] Tailor P, Tamura T, Kong HJ, et al. The feedback phase of type I interferon induction in dendritic cells requires interferon regulatory factor 8. Immunity. 2007;27(2):228–39. 17702615 10.1016/j.immuni.2007.06.009 PMC2768351

[73] Zhang D, Ding Z, Xu X. Pathologic mechanisms of the newcastle disease virus. Viruses. 2023;15(4). 10.3390/v15040864 PMC10143668 37112843

[74] Cheng J, Sun Y, Zhang X, et al. Toll-like receptor 3 inhibits Newcastle disease virus replication through activation of pro-inflammatory cytokines and the type-1 interferon pathway. Arch Virol. 2014;159(11):2937–48. 24934575 10.1007/s00705-014-2148-6

[75] Qian J, Xu X, Ding J, et al. Newcastle disease virus-like particles induce DC maturation through TLR4/NF-kappaB pathway and facilitate DC migration by CCR7-CCL19/CCL21 axis. Vet Microbiol. 2017;203:158–66. 28619138 10.1016/j.vetmic.2017.03.002

[76] Burke S, Shergold A, Elder MJ, et al. Oncolytic Newcastle disease virus activation of the innate immune response and priming of antitumor adaptive responses in vitro. Cancer Immunol Immunother. 2020;69(6):1015–27. 32088771 10.1007/s00262-020-02495-x PMC7230062

[77] Raihan J, Ahmad U, Yong YK, Eshak Z, Othman F, Ideris A. Regression of solid breast tumours in mice by Newcastle disease virus is associated with production of apoptosis related-cytokines. BMC Cancer. 2019;19(1):315. 30947706 10.1186/s12885-019-5516-5 PMC6449948

[78] Washburn B, Schirrmacher V. Human tumor cell infection by Newcastle disease virus leads to upregulation of HLA and cell adhesion molecules and to induction of interferons, chemokines and finally apoptosis. Int J Oncol. 2002;21(1):85–93. 12063554 10.3892/ijo.21.1.85

[79] Kong S, Zhang J, Wang L, et al. Mechanisms of low MHC I expression and strategies for targeting MHC I with small molecules in cancer immunotherapy. Cancer Lett. 2024;611:217432. 39730087 10.1016/j.canlet.2024.217432

[80] Zhang ZC, Shen Y, Lin YS et al. Peptide-MHC I regulatory mechanisms and intervention strategies in anti-tumor T cell immunity. Acta Pharmacol Sin. 2025. 10.1038/s41401-025-01574-y PMC12644795 40379886

[81] Svensson-Arvelund J, Cuadrado-Castano S, Pantsulaia G, et al. Expanding cross-presenting dendritic cells enhances oncolytic virotherapy and is critical for long-term anti-tumor immunity. Nat Commun. 2022;13(1):7149. 36418317 10.1038/s41467-022-34791-8 PMC9684150

[82] Evgin L, Huff AL, Wongthida P, et al. Oncolytic virus-derived type I interferon restricts CAR T cell therapy. Nat Commun. 2020;11(1):3187. 32581235 10.1038/s41467-020-17011-z PMC7314766

[83] Jung IY, Bartoszek RL, Rech AJ, et al. Type I interferon signaling via the EGR2 transcriptional regulator potentiates CAR T Cell-Intrinsic dysfunction. Cancer Discov. 2023;13(7):1636–55. 37011008 10.1158/2159-8290.CD-22-1175 PMC10330003

[84] Zamarin D, Ricca JM, Sadekova S, et al. PD-L1 in tumor microenvironment mediates resistance to oncolytic immunotherapy. J Clin Invest. 2018;128(4):1413–28. 29504948 10.1172/JCI98047 PMC5873884

[85] Yu X, Jiang H, Li J, et al. NDV inhibited IFN-β secretion through impeding CHCHD10-mediated mitochondrial fusion to promote viral proliferation. Vet Microbiol. 2024;290:109973. 38211361 10.1016/j.vetmic.2023.109973

[86] Nan FL, Zhang H, Nan WL, et al. Lentogenic NDV V protein inhibits IFN responses and represses cell apoptosis. Vet Microbiol. 2021;261:109181. 34399297 10.1016/j.vetmic.2021.109181

[87] Liu X. The Paradoxical role of IFN-γ in cancer: balancing immune activation and immune evasion. Pathol Res Pract. 2025;272:156046. 40466579 10.1016/j.prp.2025.156046

[88] Ikeda H, Old LJ, Schreiber RD. The roles of IFN gamma in protection against tumor development and cancer immunoediting. Cytokine Growth Factor Rev. 2002;13(2):95–109. 11900986 10.1016/s1359-6101(01)00038-7

[89] Alizadeh D, Wong RA, Gholamin S, et al. IFNgamma is critical for CAR T Cell-Mediated myeloid activation and induction of endogenous immunity. Cancer Discov. 2021;11(9):2248–65. 33837065 10.1158/2159-8290.CD-20-1661 PMC8561746

[90] Larson RC, Kann MC, Bailey SR, et al. CAR T cell killing requires the IFNgammaR pathway in solid but not liquid tumours. Nature. 2022;604(7906):563–70. 35418687 10.1038/s41586-022-04585-5

[91] Bailey SR, Vatsa S, Larson RC, et al. Blockade or deletion of IFNgamma reduces macrophage activation without compromising CAR T-cell function in hematologic malignancies. Blood Cancer Discov. 2022;3(2):136–53. 35015685 10.1158/2643-3230.BCD-21-0181 PMC9414118

[92] Bailey SR, Takei HN, Escobar G, et al. IFN-γ-resistant CD28 CAR T cells demonstrate increased survival, efficacy, and durability in multiple murine tumor models. Sci Transl Med. 2025;17(801):eadp8166. 40465687 10.1126/scitranslmed.adp8166 PMC12687918

[93] Pecora AL, Rizvi N, Cohen GI, et al. Phase I trial of intravenous administration of PV701, an oncolytic virus, in patients with advanced solid cancers. J Clin Oncol. 2002;20(9):2251–66. 11980996 10.1200/JCO.2002.08.042

[94] Laurie SA, Bell JC, Atkins HL, et al. A phase 1 clinical study of intravenous administration of PV701, an oncolytic virus, using two-step desensitization. Clin Cancer Res. 2006;12(8):2555–62. 16638865 10.1158/1078-0432.CCR-05-2038

[95] Hotte SJ, Lorence RM, Hirte HW, et al. An optimized clinical regimen for the oncolytic virus PV701. Clin Cancer Res. 2007;13(3):977–85. 17289893 10.1158/1078-0432.CCR-06-1817

[96] Csatary LK, Gosztonyi G, Szeberenyi J, et al. MTH-68/H oncolytic viral treatment in human high-grade gliomas. J Neurooncol. 2004;67(1–2):83–93. 15072452 10.1023/b:neon.0000021735.85511.05

[97] Freeman AI, Zakay-Rones Z, Gomori JM, et al. Phase I/II trial of intravenous NDV-HUJ oncolytic virus in recurrent glioblastoma multiforme. Mol Therapy: J Am Soc Gene Therapy. 2006;13(1):221–8. 10.1016/j.ymthe.2005.08.016 16257582

[98] Elankumaran S, Chavan V, Qiao D, et al. Type I interferon-sensitive Recombinant Newcastle disease virus for oncolytic virotherapy. J Virol. 2010;84(8):3835–44. 20147405 10.1128/JVI.01553-09 PMC2849496

[99] Shen Z, Liu X, Fan G, et al. Improving the therapeutic efficacy of oncolytic viruses for cancer: targeting macrophages. J Translational Med. 2023;21(1):842. 10.1186/s12967-023-04709-z PMC10666393 37993941

[100] Abd-Aziz N, Stanbridge EJ, Shafee N. Newcastle disease virus degrades HIF-1alpha through proteasomal pathways independent of VHL and p53. J Gen Virol. 2016;97(12):3174–82. 27902314 10.1099/jgv.0.000623 PMC5203671

[101] Yamaguchi Y, Gibson J, Ou K et al. PD-L1 blockade restores CAR T cell activity through IFN-gamma-regulation of CD163 + M2 macrophages. J Immunother Cancer. 2022;10(6). 10.1136/jitc-2021-004400 PMC9226933 35738799

[102] Kim SH, Samal SK. Newcastle disease virus as a vaccine vector for development of human and veterinary vaccines. Viruses. 2016;8(7). 10.3390/v8070183 PMC4974518 27384578

[103] Liang M. Oncorine, the world first oncolytic virus medicine and its update in China. Curr Cancer Drug Targets. 2018;18(2):171–6. 29189159 10.2174/1568009618666171129221503

[104] Frampton JE. Teserpaturev/G47Delta: first approval. BioDrugs. 2022;36(5):667–72. 36098872 10.1007/s40259-022-00553-7

[105] Pol J, Kroemer G, Galluzzi L. First oncolytic virus approved for melanoma immunotherapy. Oncoimmunology. 2016;5(1):e1115641. 26942095 10.1080/2162402X.2015.1115641 PMC4760283

[106] Frederico C, Conceição A, Nóbrega C, Mendonça LS. Advanced therapy medicinal products development - from guidelines to medicines in the market. Biotechnol Adv. 2025;83:108612. 40412771 10.1016/j.biotechadv.2025.108612

[107] FDA/CBER. Chemistry, Manufacturing, and Control (CMC) Information for Human Gene Therapy INDs 2020. (https://www.fda.gov/regulatory-information/search-fda-guidance-documents/design-and-analysis-shedding-studies-virus-or-bacteria-based-gene-therapy-and-oncolytic-products).

[108] FDA/CBER. Long-Term Follow-Up After Administration of Human Gene Therapy Products (https://www.fda.gov/regulatory-information/search-fda-guidance-documents/long-term-follow-after-administration-human-gene-therapy-products). 2020.

[109] FDA/CBER. Design and Analysis of Shedding Studies for Virus- or Bacteria-Based Gene Therapy and Oncolytic Products (https://www.fda.gov/media/113760/download). 2020.

[110] Union E. Regulation (EC) No 1394/2007 on Advanced Therapy Medicinal Products 2007. (https://eur-lex.europa.eu/eli/reg/2007/1394/oj/eng).

[111] EMA/CAT. Advanced therapy classification: CAT procedure overview 2009. (https://www.ema.europa.eu/en/human-regulatory-overview/marketing-authorisation/advanced-therapies-marketing-authorisation/advanced-therapy-classification). 10.1007/s00103-011-1309-y 21698533

[112] Union EEU. Directive 2001/18/EC (deliberate release of GMOs); directive 2009/41/EC (contained use of GMMs) 2001. (https://eur-lex.europa.eu/eli/dir/2001/18/oj/eng).

[113] Mamola JA, Chen CY, Currier MA, Cassady K, Lee DA, Cripe TP. Opportunities and challenges of combining adoptive cellular therapy with oncolytic virotherapy. Mol Ther Oncolytics. 2023;29:118–24. 37250971 10.1016/j.omto.2023.04.008 PMC10209482

[114] Rosewell Shaw A, Morita D, Porter CE, et al. IL-12 encoding oNDV synergizes with CAR-T cells in orthotopic models of non-small cell lung cancer. Mol Ther Oncol. 2024;32(4):200899. 39624055 10.1016/j.omton.2024.200899 PMC11609364

[115] Jarjour NN, Dalzell TS, Maurice NJ et al. Collaboration between interleukin-7 and – 15 enables adaptation of tissue-resident and circulating memory CD8(+) T cells to cytokine deficiency. Immunity. 2025;58(3):616–31.e5. 10.1016/j.immuni.2025.02.009 PMC13329436 40023156

[116] Steffin D, Ghatwai N, Montalbano A, et al. Interleukin-15-armoured GPC3 CAR T cells for patients with solid cancers. Nature. 2025;637(8047):940–6. 39604730 10.1038/s41586-024-08261-8 PMC12704925

[117] Torres Chavez AG, McKenna MK, Gupta A, et al. IL-7 armed binary CAR T cell strategy to augment potency against solid tumors. Front Immunol. 2025;16:1618404. 40808942 10.3389/fimmu.2025.1618404 PMC12343627

[118] Ozga AJ, Chow MT, Luster AD. Chemokines and the immune response to cancer. Immunity. 2021;54(5):859–74. 33838745 10.1016/j.immuni.2021.01.012 PMC8434759

[119] Luo H, Su J, Sun R, et al. Coexpression of IL7 and CCL21 increases efficacy of CAR-T cells in solid tumors without requiring preconditioned lymphodepletion. Clin Cancer Res. 2020;26(20):5494–505. 32816947 10.1158/1078-0432.CCR-20-0777

[120] Lin X, Kang K, Chen P, et al. Regulatory mechanisms of PD-1/PD-L1 in cancers. Mol Cancer. 2024;23(1):108. 38762484 10.1186/s12943-024-02023-w PMC11102195

[121] Cherkassky L, Morello A, Villena-Vargas J, et al. Human CAR T cells with cell-intrinsic PD-1 checkpoint Blockade resist tumor-mediated Inhibition. J Clin Invest. 2016;126(8):3130–44. 27454297 10.1172/JCI83092 PMC4966328

[122] Rafiq S, Yeku OO, Jackson HJ, et al. Targeted delivery of a PD-1-blocking ScFv by CAR-T cells enhances anti-tumor efficacy in vivo. Nat Biotechnol. 2018;36(9):847–56. 30102295 10.1038/nbt.4195 PMC6126939

[123] Chen J, Zhu T, Jiang G, Zeng Q, Li Z, Huang X. Target delivery of a PD-1-TREM2 ScFv by CAR-T cells enhances anti-tumor efficacy in colorectal cancer. Mol Cancer. 2023;22(1):131. 37563723 10.1186/s12943-023-01830-x PMC10413520

[124] Galassi C, Chan TA, Vitale I, Galluzzi L. The hallmarks of cancer immune evasion. Cancer Cell. 2024;42(11):1825–63. 39393356 10.1016/j.ccell.2024.09.010

[125] Maamary J, Array F, Gao Q, et al. Newcastle disease virus expressing a dendritic cell-targeted HIV gag protein induces a potent gag-specific immune response in mice. J Virol. 2011;85(5):2235–46. 21159873 10.1128/JVI.02036-10 PMC3067785

[126] Vigil A, Martinez O, Chua MA, Garcia-Sastre A. Recombinant Newcastle disease virus as a vaccine vector for cancer therapy. Mol Therapy: J Am Soc Gene Therapy. 2008;16(11):1883–90. 10.1038/mt.2008.181 PMC2878970 18714310

[127] Allen GM, Frankel NW, Reddy NR, et al. Synthetic cytokine circuits that drive T cells into immune-excluded tumors. Science. 2022;378(6625):eaba1624. 36520915 10.1126/science.aba1624 PMC9970000

[128] Ruffo E, Butchy AA, Tivon Y, et al. Post-translational covalent assembly of CAR and synnotch receptors for programmable antigen targeting. Nat Commun. 2023;14(1):2463. 37160880 10.1038/s41467-023-37863-5 PMC10169838

[129] Sun S, Wang X, Chen Y et al. Preclinical evaluation of antitumor activity and toxicity of TROP2-specific CAR-T cells for treatment of triple-negative breast cancer. J Immunother Cancer. 2025;13(9). 10.1136/jitc-2025-012442 PMC12410632 40903191

